# Growth promotion of three microalgae, *Chlamydomonas reinhardtii*, *Chlorella vulgaris* and *Euglena gracilis*, by in situ indigenous bacteria in wastewater effluent

**DOI:** 10.1186/s13068-018-1174-0

**Published:** 2018-06-25

**Authors:** Tadashi Toyama, Mari Kasuya, Tsubasa Hanaoka, Naoto Kobayashi, Yasuhiro Tanaka, Daisuke Inoue, Kazunari Sei, Masaaki Morikawa, Kazuhiro Mori

**Affiliations:** 1 Graduate Faculty of Interdisciplinary Research, University of Yamanashi, 4-3-11 Takeda, Kofu, Yamanashi 400-8511 Japan; 2 Division of Sustainable Energy and Environmental Engineering, Osaka University, 2-1 Yamadaoka, Suita, Osaka 565-0871 Japan; 3 Department of Health Science, Kitasato University, 1-15-1 Kitasato, Sagamihara-Minami, Kanagawa 252-0373 Japan; 4 Division of Biosphere Science, Graduate School of Environmental Science, Hokkaido University, Kita-10 Nishi-5, Kita-ku, Sapporo, 060-0810 Japan

**Keywords:** Microalgae, Wastewater effluent, Enhanced biomass production, Microalgae growth-promoting bacteria

## Abstract

**Background:**

Microalgae are a promising biomass feedstock for biofuels production. The use of wastewater effluent as a nutrient medium would improve the economics of microalgal biofuels production. Bacterial communities in aquatic environments may either stimulate or inhibit microalgal growth. Microalgal productivity could be enhanced if the positive effects of indigenous bacteria could be exploited. However, much is unknown about the effects of indigenous bacteria on microalgal growth and the characteristics of bacterial communities associated with microalgae in microalgae–effluent culture. To assess the effects of the indigenous bacteria in wastewater effluent on microalgal growth, three microalgae, *Chlamydomonas reinhardtii*, *Chlorella vulgaris*, and *Euglena gracilis*, were cultured in two municipal wastewater effluents and one swine wastewater effluent with and without indigenous bacteria for 7 days.

**Results:**

All microalgae grew better in all effluents with indigenous bacteria than without bacteria. Biomass production of *C. reinhardtii*, *C. vulgaris*, and *E. gracilis* increased > 1.5, 1.8–2.8, and > 2.1-fold, respectively, compared to the axenic cultures of each microalga. The in situ indigenous bacterial communities in the effluents therefore promoted the growth of the three microalgae during 7-day cultures. Furthermore, the total numbers of bacterial 16S rRNA genes in the 7-day microalgae–effluent cultures were 109‒793 times the initial numbers. These results suggest that the three microalgae produced and supplied organic carbon that supported bacterial growth in the effluent. At the phylum and class levels, *Proteobacteria* (*Alphaproteobacteria* and *Betaproteobacteria*) and *Bacteroidetes* (*Sphingobacteriia* and *Saprospirae*) were selectively enriched in all microalgae–effluent cultures. The enriched core bacterial families and genera were functions of the microalgal species and effluents. These results suggest that certain members of the bacterial community promote the growth of their “host” microalgal species.

**Conclusion:**

To enhance their own growth, microalgae may be able to selectively stimulate specific bacterial groups from among the in situ indigenous bacterial community found in wastewater effluent (i.e., microalgae growth-promoting bacteria: MGPB). The MGPB from effluent cultures could be used as “probiotics” to enhance microalgal growth in effluent culture. Wastewater effluent may therefore be a valuable resource, not only of nutrients, but also of MGPB to enable more efficient microalgal biomass production.

**Electronic supplementary material:**

The online version of this article (10.1186/s13068-018-1174-0) contains supplementary material, which is available to authorized users.

## Background

Microalgae have attracted extensive global attention as a promising biomass feedstock for biofuels production because of their high growth rates and high capability to accumulate lipids. The use of wastewater effluent as a nutrient medium would improve the economics and sustainability of microalgal biofuels production [1–3]. Microalgal biomass production using wastewater effluent is considered to have several advantages over production of terrestrial energy crops because there is no need for extra fertilization or irrigation, and it does not compete with food crop production or agricultural land use. Also, coupling microalgal production with wastewater treatment would turn wastewater treatment plants into net energy-producing facilities [4]. However, microalgal biofuels production is still energy intensive, costly, and then not yet economically viable. To develop more efficient microalgal biofuel production, it is necessary to enhance microalgal biomass yields in microalgae–wastewater effluent cultivation facilities.

The growth of microalgae is affected by physicochemical factors such as irradiance, temperature, nutrient concentrations, CO_2_ concentrations, and pH [5, 6]. There have been many studies aimed at enhancing microalgal growth through optimization of growth conditions, including the above-mentioned physicochemical factors [7–10]. However, the influence of indigenous microorganisms on microalgal growth has often been overlooked in microalgal biomass production using wastewater effluent.

In natural aquatic environments, interactions between microalgae and bacteria in the phycosphere [11], the region surrounding a phytoplankton or microalgal cell, are well documented [12–14]. Certain bacteria in the microalgal phycosphere can promote microalgal growth by creating a favorable microenvironment [15] and by providing nutrients [16], vitamins [17], phytohormones [18], chelators [19], or volatile organic compounds [20]. In contrast, certain bacteria can inhibit microalgal growth by lysis of microalgae [21], by producing growth-inhibiting compounds [22], or by competing with microalgae for nutrients [23]. In addition, some bacteria can initially promote microalgal growth but ultimately kill their microalgal host [24–27]. The bacterial community can therefore potentially increase or decrease the productivity of microalgae. It is important to identify the kinds of bacteria that promote microalgal growth and then to determine which types of bacteria—those with positive or those with negative effects on microalgal growth—predominate in the indigenous bacterial community in wastewater effluent.

If the positive effects of indigenous bacteria on the growth of microalgae can be controlled and used in microalgae–effluent culture in combination with physicochemical approaches to optimize microalgal biomass production, microalgal productivity could be greatly enhanced. However, knowledge about the effects of indigenous bacteria on microalgal growth and about the characteristics of the bacterial community associated with microalgae in microalgae–effluent culture is still limited. Previous studies have investigated the effects of single bacterial species on microalgal growth in microalgal culture media. Examples include the enhanced growth of *Chlorella vulgaris* by a plant growth-promoting bacterium *Azospirillum brasilense* [28], enhanced growth of *Botryococcus braunii* by *Rhizobium* sp. [29] and “*Candidatus* Phycosocius bacilliformis” [30], enhanced growth of *Tetraselmis striata* by *Pelagibaca bermudensis* and *Stappia* sp. [31], and enhanced growth of *Chlamydomonas reinhardtii*, *C. vulgaris*, *Scenedesmus* sp., and *B. braunii* by *Rhizobium* sp. [32]. To the best of our knowledge, there have been no studies clearly showing the potential for promotion of the growth of commonly used microalgae by indigenous bacterial communities in common wastewater effluent with the expectation of exploiting that potential to enhance microalgal biomass production. To develop a strategy for increasing the efficiency of microalgal biomass production systems using wastewater effluent, it will be necessary to comprehensively examine the effects of indigenous bacterial communities in various types of wastewater effluent on the growth of different microalgal species.

The main objectives of this study were therefore (i) to determine the effects of the indigenous bacteria in wastewater effluent on the growth of microalgae and (ii) to characterize the bacterial communities associated with different microalgal species growing in various wastewater effluents. In this study, we separately cultured three microalgal species, *C. reinhardtii*, *C. vulgaris*, and *Euglena gracilis*, in three different wastewater effluents: the secondary effluent from a municipal wastewater treatment plant sampled on two different dates, and the effluent from a secondary plant treating swine wastewater. Growths of the three microalgae in the three different effluents were compared in the presence and absence of the living indigenous bacterial communities. The compositions of the indigenous bacteria were analyzed by 16S rRNA gene amplicon sequencing.

## Methods

### Microalgae and their axenic culture

Axenic *C. reinhardtii* (NIES-2235), *C. vulgaris* (NIES-2172), and *E. gracilis* (NIES-48) were obtained from the Microbial Culture Collection, National Institute for Environmental Studies, Tsukuba, Japan. *C. reinhardtii* and *C. vulgaris* were cultured in C medium (150 mg/L Ca(NO_3_)_2_·4H_2_O, 100 mg/L KNO_3_, 50 mg/L β-Na_2_glycerophosphate·5H_2_O, 40 mg/L MgSO_4_·7H_2_O, 500 mg/L tris(hydroxymethyl)aminomethane, 0.1 μg/L vitamin B_12_, 0.1 μg/L biotin, 10 μg/L thiamine HCl, 3 mL/L PIV metals [1000 mg/L Na_2_EDTA·H_2_O, 196 mg/L FeCl_3_·6H_2_O, 36 mg/L MnCl_2_·4H_2_O, 10.4 mg/L ZnCl_2_, 4 mg/L CoCl_2_·6H_2_O, 2.5 mg/L Na_2_MoO_4_·H_2_O]; pH7.5). *E. gracilis* was cultured in CYP medium (C medium with 400 mg/L yeast extract and 600 mg/L polypeptone, pH 7.5). The three axenic microalgal cultures were incubated in a growth chamber at 28 ± 1 °C with fluorescent lamps at a photosynthetic photon flux density of 80 μmol m^−2^ s^−1^ and a 16-h:8-h light:dark cycle for 1 week. Every week thereafter a subculture was started by routine transfer into fresh culture medium.

### Wastewater samples

Three different wastewater samples were used in this study. Secondary municipal wastewater effluent (MW) was collected from the conventional activated sludge process of a municipal wastewater treatment plant in Kofu City, Yamanashi, Japan, on 22 November (MW1) and 15 December 2017 (MW2). Swine wastewater (SW) secondary effluent was collected from the conventional activated sludge process of a swine wastewater treatment plant in Chuo City, Yamanashi, Japan, on 22 January 2017. Table 1 shows the water quality characteristics of the three effluent samples. The effluent samples were first passed through a glass microfiber filter (pore size, 1 μm; GF/B grade; GE Healthcare UK Ltd, Buckinghamshire, England) and then a membrane filter (pore size, 0.8 μm; mixed cellulose esters membrane; Merck Millipore Ltd, Cork, Ireland). The purpose of filtering the water was to avoid, insofar as possible, the effects of organisms larger than bacteria, including microalgae and protozoa, and to focus on bacterial effects.

**Table 1:** Water qualities of initial effluent samples

Effluent sample	pH	TOC (mg/L)	Nitrogen (mg/L)			PO_4_–P (mg/L)
			NH_4_–N	NO_2_–N	NO_3_–N	
Secondary effluent of municipal wastewater 1 (MW1)	7.1	18.9	4.0	0.3	5.4	3.2
Secondary effluent of municipal wastewater 2 (MW2)	7.4	10.2	2.2	0.1	1.8	2.1
Secondary effluent of swine wastewater (SW)	7.7	56.4	57.4	2.0	3.1	23.4

*TOC* total organic carbon

### Microalgal culture in wastewater with and without living indigenous bacteria

To examine the effects on microalgal growth of indigenous bacterial communities in effluent samples, the growth of microalgae was compared in the presence and absence of living indigenous bacteria. In preliminary experiments, two types of sterilized effluents were prepared: by filtration (pore size, 0.2 μm; mixed cellulose esters membrane; Merck Millipore) or autoclaving (121 °C, 20 min). The growth levels of the three microalgae in both sterilized effluents were almost the same (Additional file 1). An effluent sample without living indigenous bacteria was therefore prepared by autoclaving (121 °C, 20 min). A 100-mL aliquot of autoclaved or unautoclaved effluent was put into a 200-mL flask. For each of the three microalgae (*C. reinhardtii*, *C. vulgaris*, and *E. gracilis*), 1 mL of subculture was inoculated into separate flasks. Three replicate flasks were prepared for each combination of microalga and effluent. All flasks were incubated in the growth chamber (28 ± 1 °C with fluorescent lamps at 80 μmol photons m^−2^ s^−1^ and a 16-h:8-h light:dark cycle) for 7 days. Because the growths of the three microalgae in the effluents with and without indigenous bacteria reached the stationary phase within 7 days, the experimental period was set to 7 days. All flasks were shaken for 1 min three times a day to disperse and aerate the microalgae. The chlorophyll concentration in each flask was measured daily as follows. One milliliter of culture was taken from each flask and centrifuged (11,000×*g*, 5 min) to recover microalgae and bacteria. Chlorophyll was then estimated spectrophotometrically after extraction in 100% methanol for 30 min [33]. Absorbance of the extract was measured at 665 nm (*A*_665_) and 650 nm (*A*_650_) with a spectrophotometer (UVmini-1240; Shimadzu Co. Ltd., Kyoto, Japan). The total chlorophyll (chlorophyll *a* + chlorophyll *b*: Chl *a *+ *b*) concentration (μg/mL) was calculated on the assumption that it was proportional to 4 × *A*_665_ + 25.5 × *A*_650_. After a 7-day incubation, the microalgal dry weight in each flask was measured as follows. From each flask, an aliquot of 50 mL of the culture was collected and vortexed for 3 min to uniformly suspend bacterial and microalgal cells. Microalgal cells were recovered using a pre-weighed GF/B filter, dried, and weighed. In the preliminary experiments, we prepared the *E. gracilis* (0.44 ± 0.05 mg-dry weight/mL) cultures with or without *Escherichia coli* (5.4 ± 2.2 × 10^6^ CFU/mL) in triplicate and measured the dry weights of *E. gracilis* in both cultures. The dry weight of *E. gracilis* (0.42 ± 0.06 mg-dry weight/mL) collected from the *E. gracilis* culture with *E. coli* by using the above method was the same as that (0.42 ± 0.03 mg-dry weight/mL) from the *E. gracilis* culture without *E. coli*. The method thus quantified the dry weight of microalgal cells with little interference from coexisting bacterial cells.

### Scanning electron microscopy of microalgal cell surfaces

For scanning electron microscopy (SEM), microalgal cells were collected by centrifugation (11,000×*g*, 5 min) and washed with sterilized C medium. Microalgal cells were then fixed with 4% osmium tetroxide solution at 4 °C for 3 h, dehydrated with a stepwise increase of ethanol from 30 to 100% at room temperature for 15 min each, and finally dried at the carbon dioxide critical point. Dried samples were coated using an Osmium Plasma Coater (OPC80T; Filgen, Nagoya, Japan) and then examined by SEM using a JEOL Scanning Microscope (JSM 6320F; JEOL Ltd., Tokyo, Japan).

### Bacterial DNA extraction

Bacterial populations and communities in the original effluents and microalgae–effluent cultures were analyzed by 16S rRNA gene quantitative PCR (qPCR) and 16S rRNA gene amplicon sequencing, respectively. To eliminate effects of microalgal chloroplast 16S rRNA gene on these bacterial 16S rRNA-based analyses, we removed microalgal cells and collected bacterial cells as follows. An aliquot of 10 mL of culture was collected from each flask, mixed with 5 mL of dispersing agent (5 mg/L sodium tripolyphosphate solution), and vortexed for 3 min to desorb bacteria from microalgal cells. The sample was then filtered through a GF/B glass microfiber filter to remove microalgal cells. The filtrate containing bacteria was again filtered through a membrane filter (pore size, 0.2 μm; mixed cellulose esters membrane; Merck Millipore) to collect bacterial cells. The total DNA of the bacteria on the membrane filter was extracted by using NucleoSpin Tissue (Takara Bio Inc., Shiga, Japan) according to the manufacturer’s protocol.

### Quantification of bacterial 16S rRNA gene

The bacterial 16S rRNA gene was quantified by qPCR using a set of universal primers, 341F (5ʹ-CCTACGGGAGGCAGCAG-3ʹ) and 534R (5ʹ-TACCGCGGCTGCTGGCAC-3ʹ) [34], SYBR Premix Ex Taq II (Takara Bio), and a Thermal Cycler Dice RealTime System II, model TP900/960 (Takara Bio). The qPCR temperature program included an initial denaturation at 95 °C for 1 min, followed by 40 cycles of 95 °C for 5 s, annealing at 60 °C for 30 s, and extension at 72 °C for 30 s. A standard curve for the 16S rRNA gene was created by using a custom-synthesized plasmid carrying the 16S rRNA gene sequence of *E. coli*. The qPCRs were performed in triplicate.

### Phylogenetic analysis of the bacterial community

The extracted bacterial DNA samples were subjected to Illumina MiSeq 16S rRNA gene sequencing. The V4 region of the 16S rRNA gene was amplified by PCR using universal primers 515F (5′-Seq A-TGT GCC AGC MGC CGC GGT AA-3′) and 806R (5′-Seq B-GGA CTA CHV GGG TWT CTA AT-3′). The nucleotide sequences Seq A (ACACTCTTTCCCTACACGACGCTCTTCCGATCT) and Seq B (GTGACTGGAGTTCAGACGTGTGCTCTTCCGATCT) represent the sequences targeted by the second set of PCR primers described below. The first PCR program was as follows: an initial denaturing at 94 °C for 2 min; 20 cycles of 94 °C for 30 s, 50 °C for 30 s, and 72 °C for 30 s; and an extension at 72 °C for 5 min. Fragments of 16S rDNA in the products of the first PCR were amplified again using the second PCR forward (5′-adaptor C-tag sequence Seq A-3′) and reverse primers (5′-adaptor D-Seq B-3′), where adaptors C and D were used for the MiSeq sequencing reaction. The tag sequence included eight nucleotides designed for sample identification bar coding. The second PCR program was as follows: an initial denaturing at 94 °C for 2 min; 8 cycles of 94 °C for 30 s, 60 °C for 30 s, and 72 °C for 30 s; and an extension at 72 °C for 5 min. PCR amplicons were sequenced using an Illumina MiSeq Sequencer. Sequence reads were analyzed using sickle (ver 1.33), Fastx toolkit (ver 0.0.13.2), FLASH (ver 1.2.10), and USEARCH (ver 8.0.1623_i86linux64). These analyses involved the formation of contigs, removal of error sequences, and removal of chimeras. All operational taxonomic units (OTUs) were clustered at a cutoff of 0.03 (97% similarity). Sequencing and sequence-read analyses were conducted in FASMAC (Kanagawa, Japan). Shannon diversity and principal coordinate analysis (PCoA) were analyzed using Qiime ver 1.9.0 [35]. Heatmap clustering was analyzed using R ver 2.15.2 [36].

### Statistical analysis

Each value used in the statistical analysis represented the results from three replicate samples per experiment. Each result was expressed as a mean ± SD. Significance (*P* < 0.05) was analyzed by using the *t* test in SPSS Statistics v. 22.0 (IBM, Armonk, NY, USA).

## Results and discussion

### Growth and biomass production of *C. reinhardtii*, *C. vulgaris*, and *E. gracilis* in wastewater effluent with and without indigenous bacteria

**Fig. 1:**
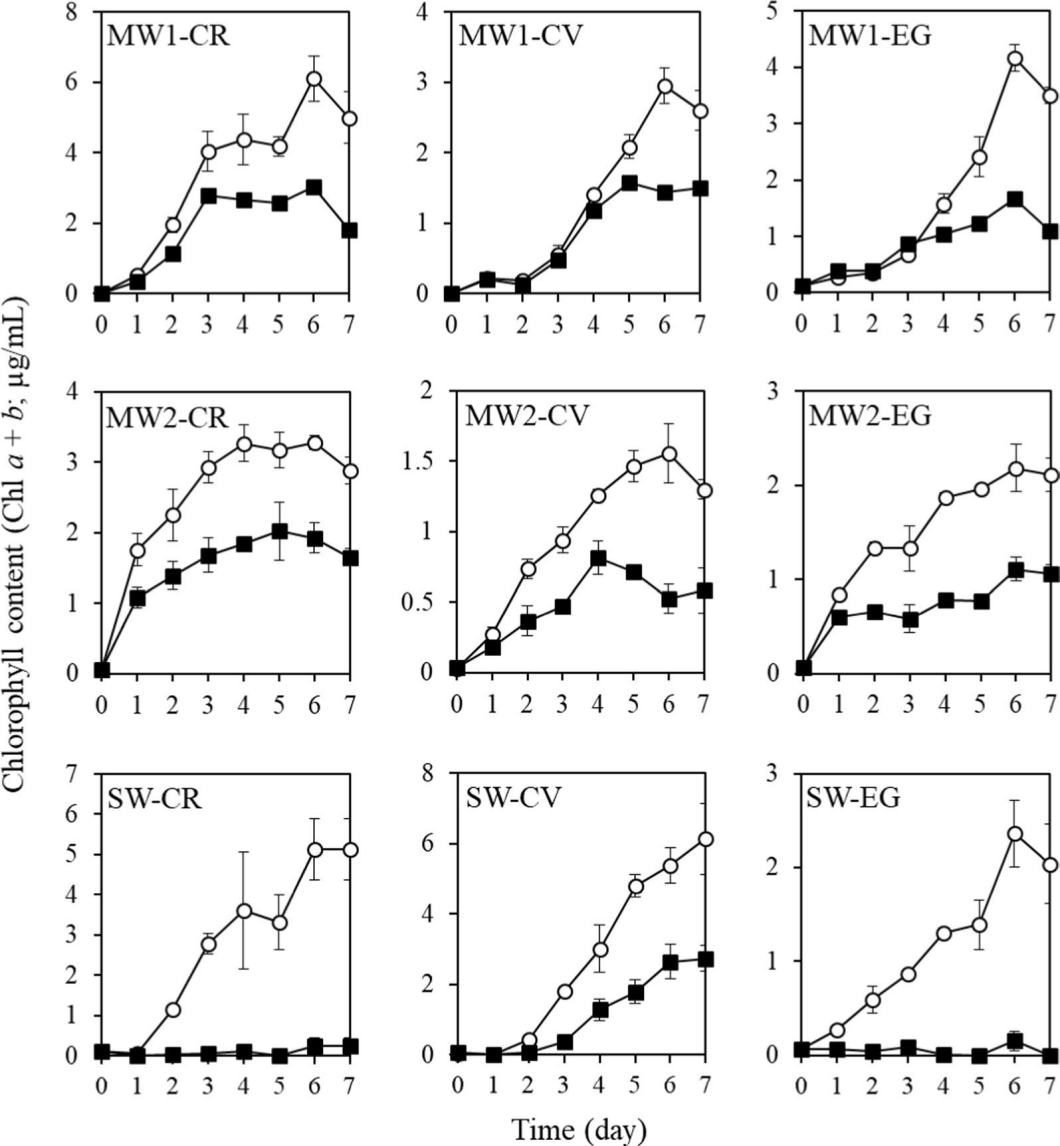
Changes in chlorophyll *a *+ *b* content in microalgal cultures with indigenous bacteria (open circles) and without indigenous bacteria (closed squares) over 7 days. Values are means ± SDs (*n* = 3). *MW1* municipal wastewater effluent 1, *MW2* municipal wastewater effluent 2, *SW* swine wastewater effluent, *CR Chlamydomonas reinhardtii*, *CV Chlorella vulgaris*, *EG Euglena gracilis*

**Fig. 2:**
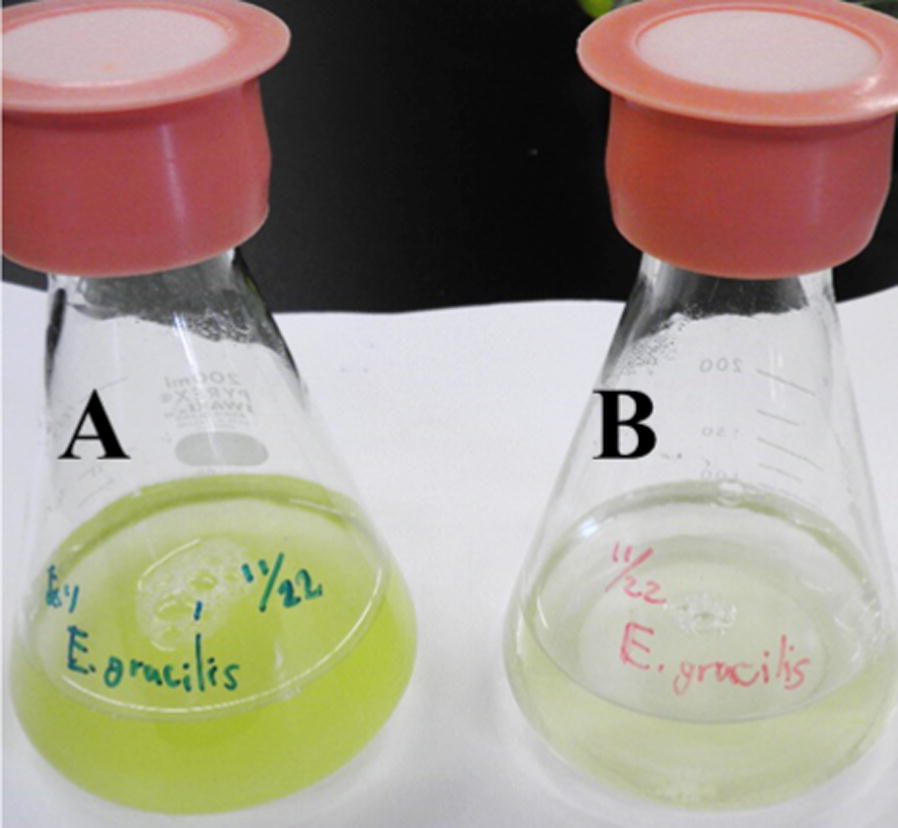
Effect of indigenous living bacteria on growth of *Euglena gracilis* in municipal wastewater (MW) effluent. Representative photograph of *E. gracilis* in MW1 effluent with living bacteria (**A**) and without living bacteria (**B**) after 7 days

To examine the effects of indigenous bacteria in wastewater effluents on the growth of the microalgae *C. reinhardtii*, *C. vulgaris*, and *E. gracilis*, each microalgal species was grown separately in the three effluents with or without indigenous bacteria for 7 days. The chlorophyll concentrations were significantly higher in the cultures with bacteria than in the axenic microalgal cultures (*P* < 0.05) (Figs. 1 and 2). In the axenic microalgal cultures, chlorophyll concentrations reached maximal levels and stopped increasing within 3–6 days. *C. reinhardtii* and *E. gracilis* did not grow in the axenic SW effluent. In contrast, the chlorophyll concentrations in the cultures with bacteria tended to increase continuously until the end of the culture period. *C. reinhardtii* and *E. gracilis* grew remarkably in SW effluent with bacteria. The biomass production of *C. reinhardtii*, *C. vulgaris*, and *E. gracilis* during the 7-day culture experiment increased > 1.5, 1.8–2.8, and > 2.1-fold, respectively, compared to the axenic cultures of each microalga (Table 2). These results strongly indicate that indigenous bacterial communities in the effluents promoted the growth of the three microalgae or provided the microalgal partners with an essential compound.

**Table 2:** Microalgal biomass production in microalgae–effluent culture with and without indigenous bacteria

Effluent sample	Increase in microbial dry weight over 7 days (mg-dry weight/100 mL) and ratio of biomass of indicated species of microalga with bacteria to that without bacteria		
	*Chlamydomonas reinhardtii*	*Chlorella vulgaris*	*Euglena gracilis*
MW1			
With bacteria	42.0 ± 2.6	13.8 ± 3.4	24.4 ± 2.3
Without bacteria	27.7 ± 6.4	7.5 ± 0.6	11.8 ± 0.6
With bacteria/without bacteria ratio	1.5	1.8	2.1
MW2			
With bacteria	29.1 ± 4.7	8.6 ± 0.5	12.8 ± 5.5
Without bacteria	18.7 ± 2.5	3.6 ± 1.7	3.7 ± 1.3
With bacteria/without bacteria ratio	1.6	2.4	3.5
SW			
With bacteria	42.8 ± 2.1	47.1 ± 5.1	19.0 ± 3.6
Without bacteria	ND	16.8 ± 4.5	ND
With bacteria/without bacteria ratio	IC	2.8	IC

Values are means ± SDs (*n* = 3)

*MW1* municipal wastewater effluent sample 1, *MW2* municipal wastewater effluent sample 2, *SW* swine wastewater effluent, *ND* not detectable, *IC* incalculable

Bacterial communities in aquatic environments influence the growth of microalgae by both stimulatory and inhibitory effects [37]. In our 7-day culture experiments, interestingly, the promotion of microalgal growth by indigenous bacterial communities in effluent was observed for all combinations of the three different wastewater effluents and three different microalgal species. In contrast, no inhibitory effect on microalgal growth by the indigenous bacterial communities in the effluents was observed at least in 7-day culture experiment. We therefore hypothesized that microalgae growth-promoting bacteria (MGPB) are likely present in most wastewater effluents. Previously, MGPB have been isolated from seawater [37, 38], the phycospheres of continuous lab cultures of microalgae [15, 29‒32, 39], and the rhizosphere of terrestrial plants [28]. For example, “*Ca.* Phycosocius bacilliformis” BOTRYCO-2 enhanced the biomass productivity of *B. braunii* by 1.8-fold [30], and *P. bermudensis* KCTC 13073BP increased the biomass productivity of *T. striata* twofold [31]. In our study, indigenous bacterial communities in the three effluents stimulated the growth of the three microalgal species to the same extent as previously reported for MGPB. These results suggest that, in addition to phycospheres and rhizospheres, wastewater effluent could be a universal source of effective MGPB for various microalgal species. Although we cannot rule out the possibility that microalga essentially require some common products of environmental bacteria, wastewater effluent can thus be a valuable resource, not only of nutrients [1–3], but also of MGPB to enhance microalgal biomass productivity. Several reports are available on not only the growth but also the change in cellular composition and flocculation of microalga when co-cultured with bacteria [20, 30, 39–41]. In this study, lipid productivity was not considered and this still remaining as a future task. However, it may be worth to note that in most cases the content of lipids [20, 39, 41] and hydrocarbons [30] in microalgae increases by their associated bacteria. On the other hand, long-term effects of indigenous bacterial communities in effluents on microalgal growth must be examined in future studies because some bacteria can initially promote microalgal growth but eventually kill their microalgal host [24–27].

### Changes in the bacterial population in the cultures

**Fig. 3:**
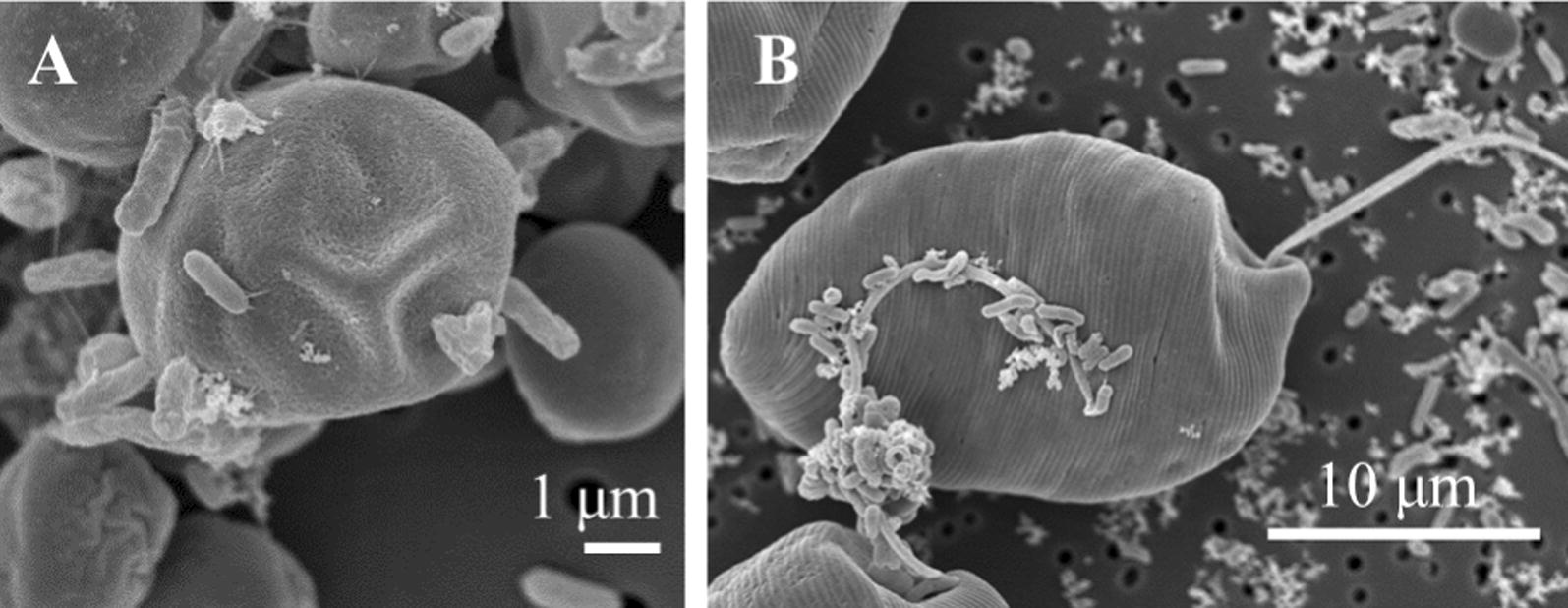
SEM images of bacteria attached to *Chlorella vulgaris* cells (**A**) and *Euglena gracilis* cells (**B**) cultured in MW1 with indigenous bacteria after 7 days

Observations by SEM (Fig. 3) revealed that several bacterial species attached to the microalgal cells in the culture over 7 days. Although it was probable that not all of these bacteria were recovered, the free-living bacteria in the microalgae–effluent culture and the bacteria attached onto microalgal cells were collected as above described in ‘Method’ section. The total number of bacterial 16S rRNA genes significantly increased (*P* < 0.05) in all the cultures over 7 days; by 148‒219, 109‒327, and 333‒793-fold compared with the initial values in microalgae-MW1, -MW2, and -SW, respectively (Table 3). These results suggest that the three microalgae produced and supplied organic carbon into the phycosphere and bulk water that effectively supported bacterial growth [42, 43].

**Table 3:** Quantification of bacterial 16S rRNA genes in initial effluent and 7-day cultures

Effluent sample	16S rRNA gene copy numbers (copies/mL), and their ratios in initial effluent and in 7-day cultures			
	Initial	After cultivation with indicated microalgal species		
		*Chlamydomonas reinhardtii*	*Chlorella vulgaris*	*Euglena gracilis*
MW1 effluent	4.2 ± 1.1 × 10^8^	9.2 ± 2.5 × 10^10^	6.2 ± 3.6 × 10^10^	7.4 ± 1.7 × 10^10^
7-day culture/initial ratio		219	148	176
MW2 effluent	2.2 ± 0.9 × 10^8^	7.2 ± 2.9 × 10^10^	4.7 ± 1.9 × 10^10^	2.4 ± 1.1 × 10^10^
7-day culture/initial ratio		327	214	109
SW effluent	8.7 ± 3.3 × 10^9^	6.9 ± 5.2 × 10^12^	6.5 ± 3.1 × 10^12^	2.9 ± 1.5 × 10^12^
7-day culture/initial ratio		793	747	333

Values are means ± SDs (*n* = 3)

*MW1* municipal wastewater effluent sample 1, *MW2* municipal wastewater effluent sample 2, *SW* swine wastewater effluent

### Bacterial community composition in the cultures

The bacterial communities in the effluent samples before and after 7 day of culture with each microalgal species were analyzed by comparison of the V4 region of the 16S rRNA gene sequences (Table 4). The Shannon index and OTU count were lower in microalgae–effluent cultures than in the original effluents, except for the OTU of *C. vulgaris*-MW2. This result suggests that microalgae exerted a selective pressure on the effluent bacterial communities during the 7 days of culture.

**Table 4:** Read number, number of operational taxonomic units (OTUs), and Shannon index of bacterial communities in initial effluent and 7-day cultures

Sample	Filtered reads	OTUs	Shannon index
MW1	113,724	6932	9.15
MW1-CR	100,924	4498	3.51
MW1-CV	103,972	3599	3.98
MW1-EG	119,590	5007	4.23
MW2	127,497	6354	8.09
MW2-CR	106,184	4500	4.28
MW2-CV	124,432	6558	4.85
MW2-EG	109,082	4563	4.57
SW	88,950	6226	7.62
SW-CR	123,023	4108	4.78
SW-CV	117,494	4572	4.75
SW-EG	256,138	4211	5.90

*MW1* municipal wastewater effluent sample 1, *MW2* municipal wastewater effluent sample 2, *SW* swine wastewater effluent, *CR Chlamydomonas reinhardtii*, *CV Chlorella vulgaris*, *EG Euglena gracilis*

**Fig. 4:**
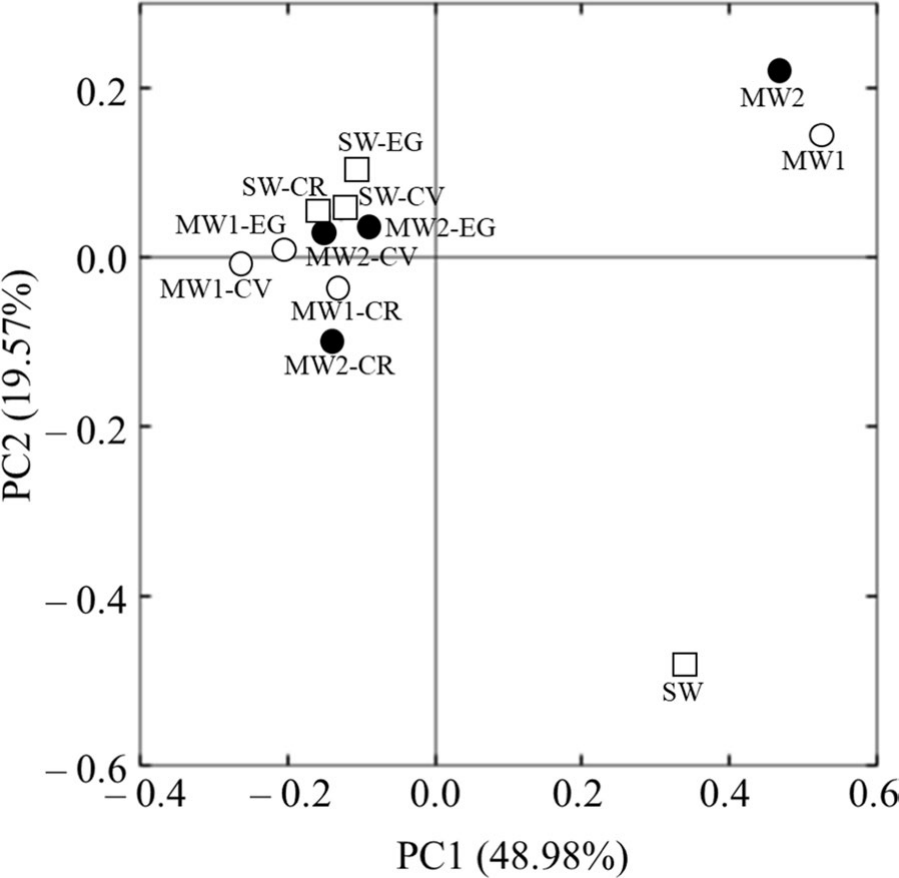
Principal coordinate analysis (PCoA) of pairwise Bray–Curtis dissimilarity index between all samples. MW1 (white circle), municipal wastewater effluent 1; MW2 (black circle), municipal wastewater effluent 2; SW (white square), swine wastewater effluent; CR, *Chlamydomonas reinhardtii*; CV, *Chlorella vulgaris*; EG, *Euglena gracilis*

**Fig. 5:**
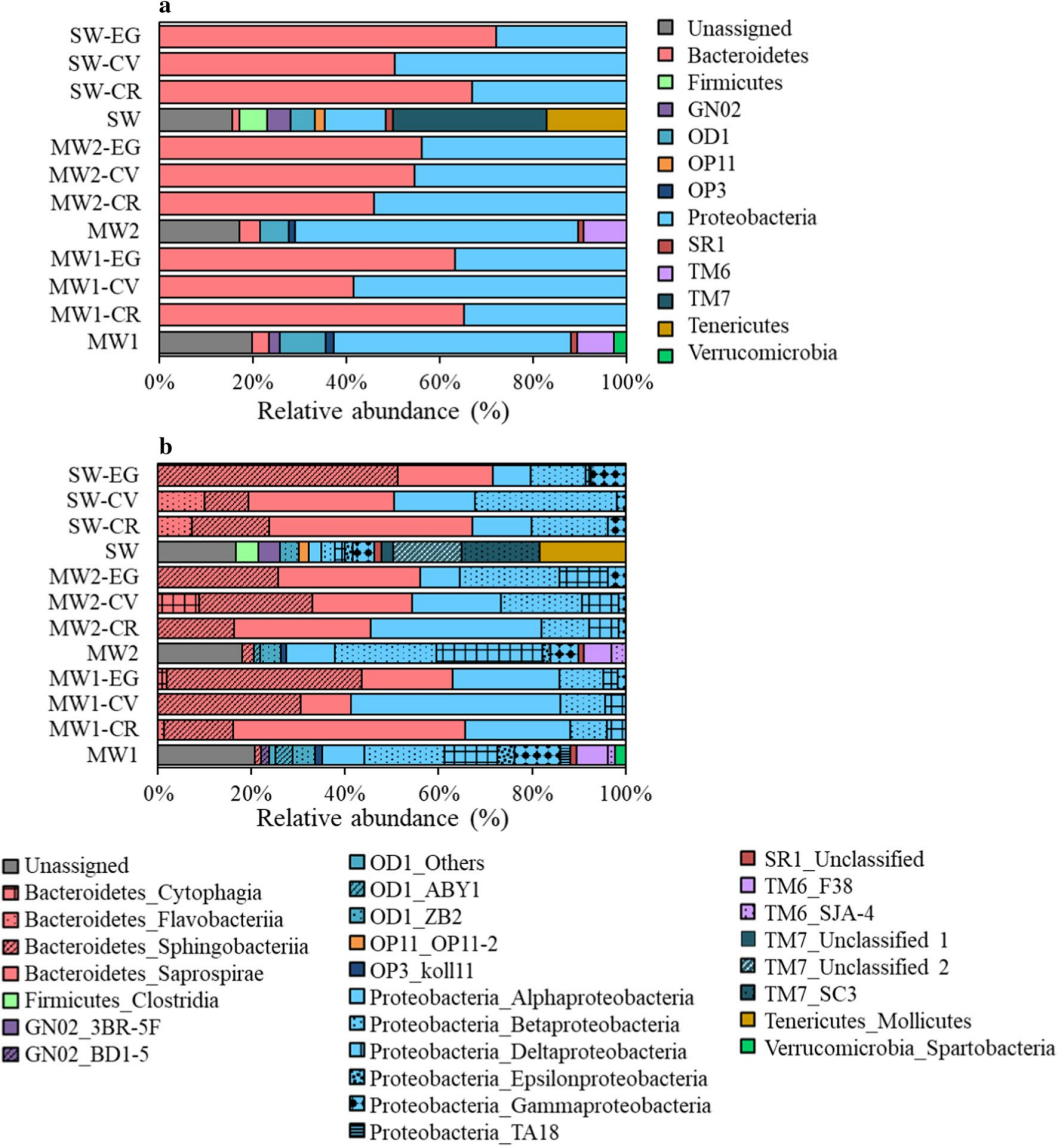
Bacterial community composition at the phylum (**a**) and class (**b**) levels. *MW1* municipal wastewater effluent 1, *MW2* municipal wastewater effluent 2, *SW* swine wastewater effluent, *CR Chlamydomonas reinhardtii*, *CV Chlorella vulgaris*, *EG Euglena gracilis*

We used PCoA to visualize the differences in the relative abundances of OTUs in each bacterial community (Fig. 4). There were clear differences between the bacterial communities in each effluent before and after cultivation with each microalga. Interestingly, after the 7-day culture, the bacterial communities associated with each microalga were clustered closer to each other, even though the original effluents and microalgal species differed. The bacterial community compositions were further examined at both the phylum and class levels (Fig. 5). At the phylum level (Fig. 5a), the initial bacterial communities in MW1 and MW2 effluents were dominated by *Proteobacteria* (46.9 and 56.6% of total phylum groups, respectively). *Proteobacteria* (12.5%), Candidate division TM7 (31.5%), and *Tenericutes* (16.5%) were dominant in the SW effluent. In contrast, *Proteobacteria* (26.6–57.9%) and *Bacteroidetes* (35.3–68.4%) were dominant in the bacterial communities associated with microalgae after the 7-day culture. At the class level (Fig. 5b), *Sphingobacteriia* (8.9–48.3%), *Saprospirae* (10.4–47.4%), *Alphaproteobacteria* (7.1–43.7%), and *Betaproteobacteria* (7.6–28.9%) were most abundant in the bacterial communities associated with all microalgal cultures. The relative abundances of these four bacterial classes in the cultures were clearly higher than in the initial effluents. In contrast, the relative abundances of *Deltaproteobacteria* and *Gammaproteobacteria* tended to be lower in cultures than in the initial effluents.

*Sphingobacteriia*, *Alphaproteobacteria*, and *Betaproteobacteria* have been generally acknowledged to be the dominant bacterial members in phycospheres of marine microalgae [44], freshwater microalgae [45], wastewater-based microalgal ponds [46–48], and photobioreactors [49]. In this study, the similar selection of *Bacteroidetes* (*Sphingobacteriia* and *Saprospirae*) and *Proteobacteria* (*Alphaproteobacteria* and *Betaproteobacteria*) in all microalgae-effluent cultures of the three microalgal species and three different effluents was observed after culturing for only 7 days.

**Fig. 6:**
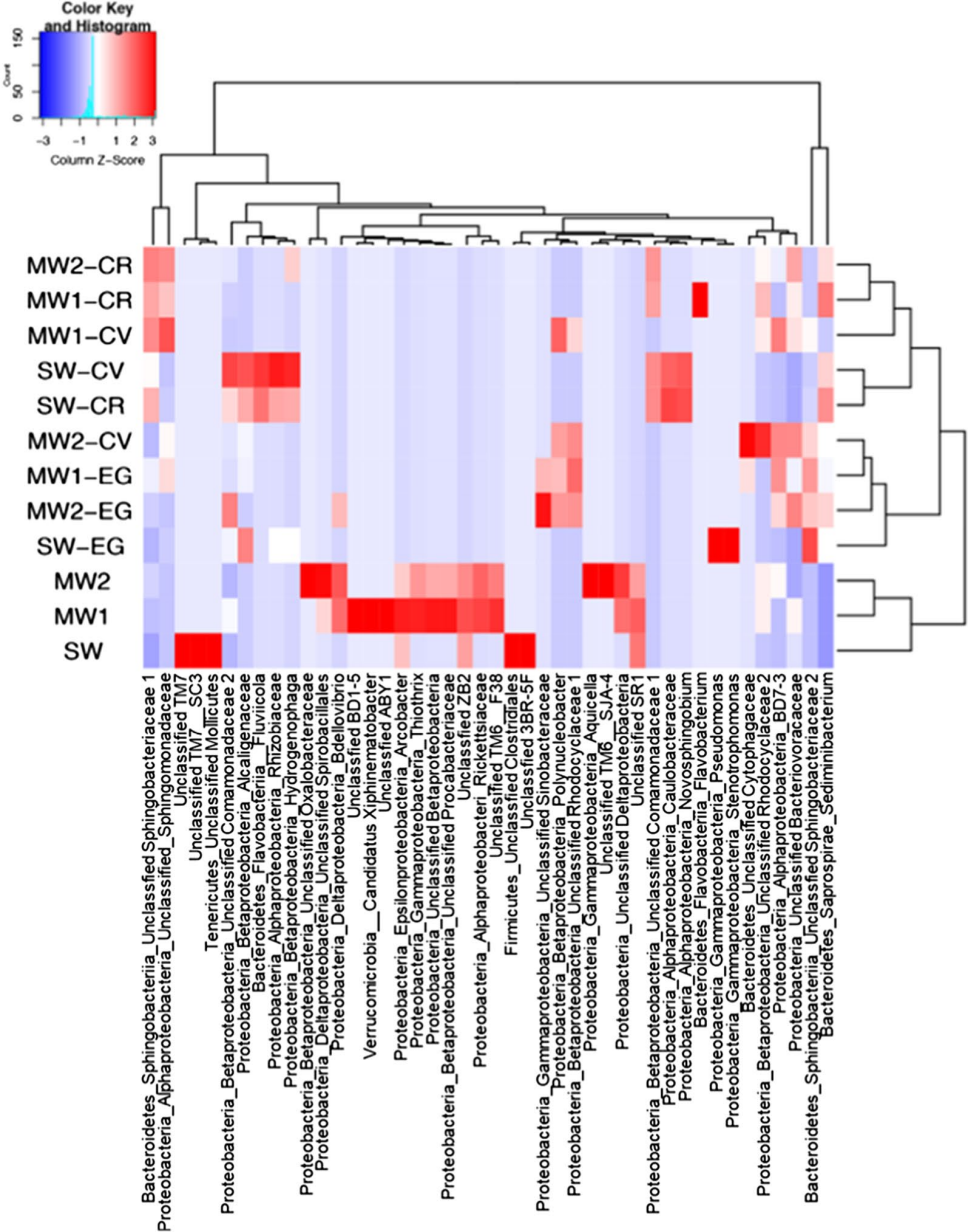
Heatmap showing the most abundant family or genera in all samples. Heatmap was constructed from the Bray–Curtis dissimilarity matrix using genera (> 1% relative abundance). Cluster dendrograms are based on average linkage hierarchical clustering. *MW1* municipal wastewater effluent 1, *MW2* municipal wastewater effluent 2, *SW* swine wastewater effluent, *CR Chlamydomonas reinhardtii*, *CV Chlorella vulgaris*, *EG Euglena gracilis*

Hierarchically clustered heatmap analysis was used to identify the core bacterial groups at the family or genus level (Fig. 6). There were clear differences between bacterial communities before and after cultivation with each microalga. *Sphingobacteriaceae*, *Cytophagaceae*, *Fluviicola*, or *Sediminibacterium* within the *Bacteroidetes* phylum; *Sphingomonadaceae*, *Rhizobiaceae*, *Caulobacteraceae*, or *Novosphingobium* within the *Alphaproteobacteria* phylum; and *Alcaligenaceae*, *Rhodocyclaceae*, *Comamonadaceae*, *Hydrogenophaga*, and *Polynucleobacter* within the *Betaproteobacteria* phylum were the dominant family or genus members in the bacterial communities associated with microalgae. These core members of the bacterial communities may have strongly impacted the growth of the microalgae. Furthermore, except for the *C. vulgaris*-MW2 culture, the bacterial communities in the culture with each microalga were clearly divided into two distinctly different main clusters: (i) *C. reinhardtii* and *C. vulgaris* cultures, and (ii) *E. gracilis* cultures. In addition, the core bacterial members in cultures depended on the combination of microalgal species and effluent. For example, the core bacteria in *C. reinhardtii*, *C. vulgaris*, and *E. gracilis* cultures grown in MW1 effluent differed from each other. These findings suggest that each microalgal species exerted selective pressure on the indigenous bacteria, and this pressure likely resulted from organic carbon metabolites unique to each microalgal species that effectively and selectively supported bacterial growth [42, 50, 51]. *C. reinhardtii* and *C. vulgaris* are members of the *Chlorophyta* (green algae), whereas *E. gracilis* belongs to the *Euglenozoa*. Organic carbon metabolites of *C. reinhardtii* and *C. vulgaris* might be similar to each other. This similarity might be related to the relatively similar bacterial core members associated with *C. reinhardtii* and *C. vulgaris*.

A noteworthy new finding from this study was that promotion of the growth of the three microalgal species by the indigenous bacterial community was observed for all of the wastewater effluents. Although the possibility cannot be denied that certain bacteria promoted the growth of all three microalgae, our results strongly suggest that within the bacterial community certain species promote the growth of their “host” microalgal species, and that each microalgal species recruit these species-specific MGPB from the bacterial community in the effluents. Further studies are needed to examine the species-specific interactions of bacteria and microalgae. Isolation and identification of the MGPB strains and clarification of their microalgal growth-promoting functions are ongoing. Isolation of MGPB for each microalgal species from effluent cultures could lead to their use as “probiotics” to enhance microalgal growth effectively in effluent culture.

## Conclusions

Indigenous bacterial communities in three different wastewater effluents—two from municipal wastewater and one from swine wastewater—significantly promoted the growth of three microalgae, *C. reinhardtii*, *C. vulgaris*, and *E. gracilis* during 7-day cultures. The fact that similar growth promotion was observed for all combinations of the effluents and microalgal species supports the conclusion that MGPB are ubiquitously present in a wide variety of wastewater effluents. An important result of this study was the discovery that wastewater effluent can be used as a microalgal culture platform for highly efficient biomass production enabled by MGPB. This insight will provide a stimulus for microalgal biomass production using wastewater effluent.

## Additional file

**Additional file 1.** Changes in chlorophyll a + b content in microalgal cultures using the two sterilized secondary municipal wastewater effluent samples: filtered (pore size, 0.2 μm) effluent (closed circles) and autoclaved (121 °C, 20 min) effluent (open circles). Values are means ± SDs (*n *= 3).

## Abbreviations

MGPB: microalgae growth-promoting bacteria
CFU: colony-forming units
SEM: scanning electron microscopy
DNA: deoxyribonucleic acid
rRNA: ribosomal ribonucleic acid
PCR: polymerase chain reaction
OTUs: operational taxonomic units
PCoA: principal coordinate analysis
SD: standard deviation
