# Supplementary material for: Growth promotion of three microalgae, Chlamydomonas reinhardtii, Chlorella vulgaris and Euglena gracilis, by in situ indigenous bacteria in wastewater effluent
Source: Biotechnol Biofuels. 2018 Jun 25;11:176. doi: 10.1186/s13068-018-1174-0 (PMC6016138; doi:10.1186/s13068-018-1174-0)
Supplement: Supplementary file 1 — Additional file 1. Changes in chlorophyll a + b content in microalgal cultures using the two sterilized secondary municipal wastewater effluent samples: filtered (pore size, 0.2 μm) effluent (closed circles) and autoclaved (121 °C, 20 min) effluent (open circles). Values are means ± SDs (n = 3). [file 13068_2018_1174_MOESM1_ESM.pdf]

Additional file 1

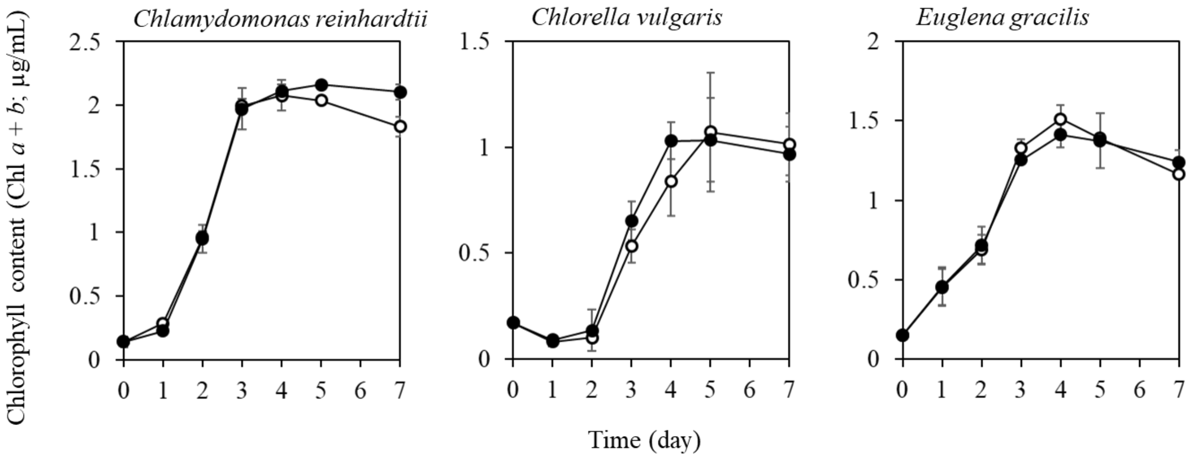


Changes in chlorophyll a + b content in microalgal cultures using the two sterilized secondary municipal wastewater effluent samples: filtrated (pore size, 0.2 μm) effluent (closed circles) and autoclaved (121 °C, 20 min) effluent (open circles). Values are means ± SDs (*n*=3).
